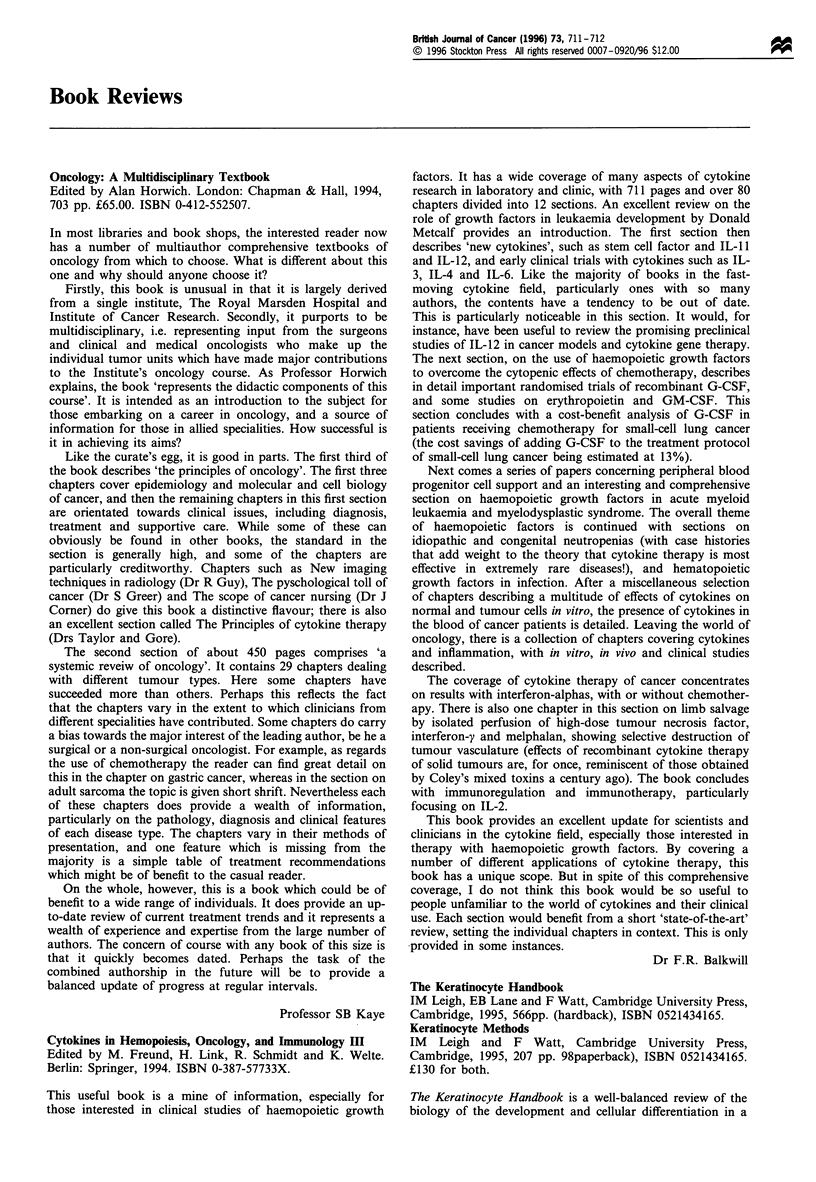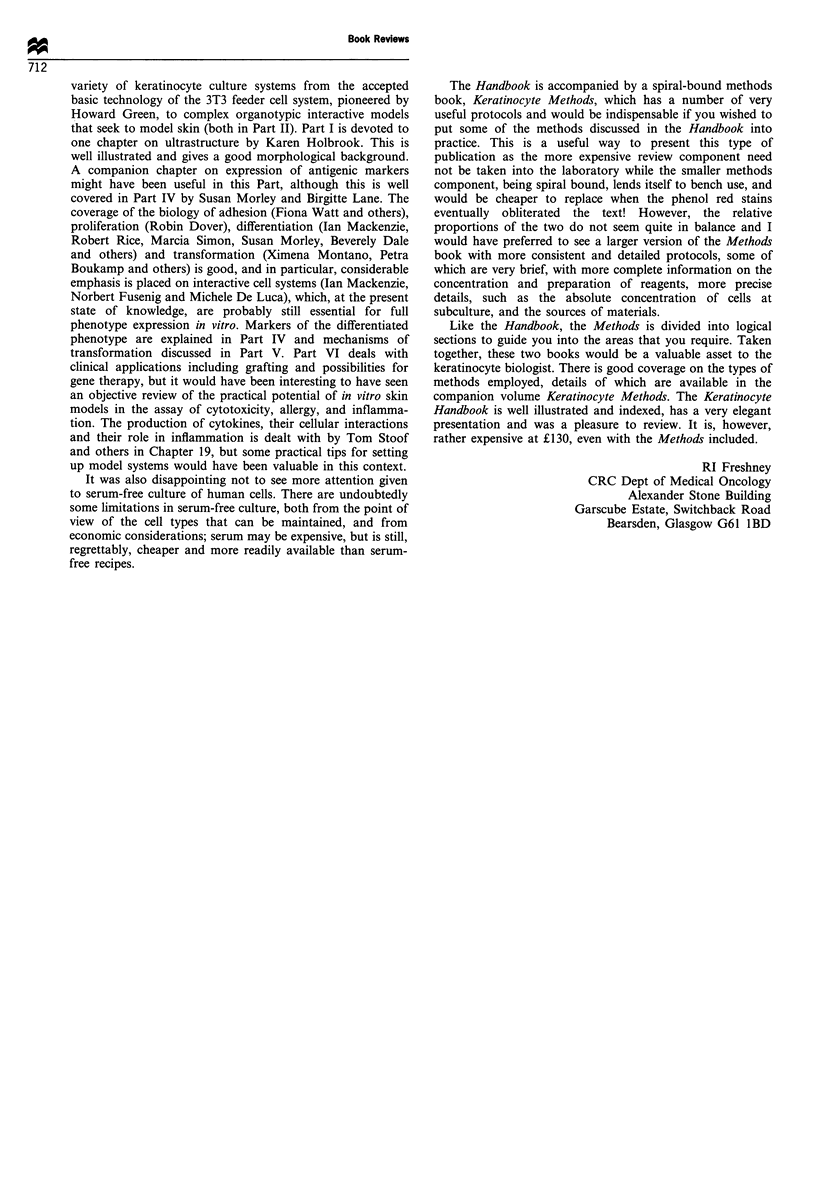# The Keratinocyte Handbook

**Published:** 1996-03

**Authors:** RI Freshney


**The Keratinocyte Handbook**
IM Leigh, EB Lane and F Watt, Cambridge University Press, Cambridge, 1995, 566pp. (hardback), ISBN 0521434165.
**Keratinocyte Methods**
IM Leigh and F Watt, Cambridge University Press, Cambridge, 1995, 207 pp. 98paperback), ISBN 0521434165. £130 for both.

*The Keratinocyte Handbook* is a well-balanced review of the biology of the development and cellular differentiation in a variety of keratinocyte culture systems from the accepted basic technology of the 3T3 feeder cell system, pioneered by Howard Green, to complex organotypic interactive models that seek to model skin (both in Part II). Part I is devoted to one chapter on ultrastructure by Karen Holbrook. This is well illustrated and gives a good morphological background. A companion chapter on expression of antigenic markers might have been useful in this Part, although this is well covered in Part IV by Susan Morley and Birgitte Lane. The coverage of the biology of adhesion (Fiona Watt and others), proliferation (Robin Dover), differentiation (Ian Mackenzie, Robert Rice, Marcia Simon, Susan Morley, Beverely Dale and others) and transformation (Ximena Montano, Petra Boukamp and others) is good, and in particular, considerable emphasis is placed on interactive cell systems (Ian Mackenzie, Norbert Fusenig and Michele De Luca), which, at the present state of knowledge, are probably still essential for full phenotype expression *in vitro*. Markers of the differentiated phenotype are explained in Part IV and mechanisms of transformation discussed in Part V. Part VI deals with clinical applications including grafting and possibilities for gene therapy, but it would have been interesting to have seen an objective review of the practical potential of *in vitro* skin models in the assay of cytotoxicity, allergy, and inflammation. The production of cytokines, their cellular interactions and their role in inflammation is dealt with by Tom Stoof and others in Chapter 19, but some practical tips for setting up model systems would have been valuable in this context.

It was also disappointing not to see more attention given to serum-free culture of human cells. There are undoubtedly some limitations in serum-free culture, both from the point of view of the cell types that can be maintained, and from economic considerations; serum may be expensive, but is still, regrettably, cheaper and more readily available than serum-free recipes.

The *Handbook* is accompanied by a spiral-bound methods book, *Keratinocyte Methods*, which has a number of very useful protocols and would be indispensable if you wished to put some of the methods discussed in the *Handbook* into practice. This is a useful way to present this type of publication as the more expensive review component need not be taken into the laboratory while the smaller methods component, being spiral bound, lends itself to bench use, and would be cheaper to replace when the phenol red stains eventually obliterated the text! However, the relative proportions of the two do not seem quite in balance and I would have preferred to see a larger version of the *Methods* book with more consistent and detailed protocols, some of which are very brief, with more complete information on the concentration and preparation of reagents, more precise details, such as the absolute concentration of cells at subculture, and the sources of materials.

Like the *Handbook*, the *Methods* is divided into logical sections to guide you into the areas that you require. Taken together, these two books would be a valuable asset to the keratinocyte biologist. There is good coverage on the types of methods employed, details of which are available in the companion volume *Keratinocyte Methods*. The *Keratinocyte Handbook* is well illustrated and indexed, has a very elegant presentation and was a pleasure to review. It is, however, rather expensive at £130, even with the *Methods* included.

RI Freshney
CRC Dept of Medical Oncology
Alexander Stone Building
Garscube Estate, Switchback Road
Bearsden, Glasgow G61 1BD